# Antibacterial Properties and Mechanism of Activity of a Novel Silver-Stabilized Hydrogen Peroxide

**DOI:** 10.1371/journal.pone.0131345

**Published:** 2015-07-08

**Authors:** Nancy L. Martin, Paul Bass, Steven N. Liss

**Affiliations:** 1 Department of Biomedical and Molecular Sciences, Queen's University, Kingston, Ontario, Canada; 2 School of Environmental Studies, Queen's University, Kingston, Ontario, Canada; 3 Department of Chemical Engineering, Queen's University, Kingston, Ontario, Canada; NERC Centre for Ecology & Hydrology, UNITED KINGDOM

## Abstract

Huwa-San peroxide (hydrogen peroxide; HSP) is a NSF Standard 60 (maximum 8mg/L^-1^) new generation peroxide stabilized with ionic silver suitable for continuous disinfection of potable water. Experiments were undertaken to examine the mechanism of HSP against planktonic and biofilm cultures of indicator bacterial strains. Contact/kill time (CT) relationships that achieve effective control were explored to determine the potential utility in primary disinfection. Inhibitory assays were conducted using both nutrient rich media and a medium based on synthetic wastewater. Assays were compared for exposures to three disinfectants (HSP, laboratory grade hydrogen peroxide (HP) and sodium hypochlorite) at concentrations of 20 ppm (therefore at 2.5 and 5 times the NSF limit for HP and sodium hypochlorite, respectively) and at pH 7.0 and 8.5 in dechlorinated tap water. HSP was found to be more or equally effective as hypochlorite or HP. Results from CT assays comparing HSP and HP at different bacterial concentrations with neutralization of residual peroxide with catalase suggested that at a high bacterial concentration HSP, but not HP, was protected from catalase degradation possibly through sequestration by bacterial cells. Consistent with this hypothesis, at a low bacterial cell density residual HSP was more effectively neutralized as less HSP was associated with bacteria and therefore accessible to catalase. Silver in HSP may facilitate this association through electrostatic interactions at the cell surface. This was supported by experiments where the addition of mono (K^+^) and divalent (Ca^+2^) cations (0.005-0.05M) reduced the killing efficacy of HSP but not HP. Experiments designed to distinguish any inhibitory effect of silver from that of peroxide in HSP were carried out by monitoring the metabolic activity of established *P*. *aeruginosa* PAO1 biofilms. Concentrations of 70-500 ppm HSP had a pronounced effect on metabolic activity while the equivalent concentrations of ionic silver (50- 375 ppb) had a negligible effect, demonstrating that the microbiocidal activity of HSP was due to peroxide rather than silver. Overall, it was found that the antimicrobial activity of HSP is enhanced over that of hydrogen peroxide; the presence of the ionic silver enhances interactions of HSP with the bacterial cell surface rather than acting directly as a biocide at the tested concentrations.

## Introduction

Chlorine or sodium hypochlorite (NaOCl) has long been used as an effective disinfectant for drinking water [1], its use first coming into practice during the mid 1880s to help deal with typhoid fever epidemics in the port of Pola on the Adriatic Sea and in Maidstone, England [2]. Since that time, owing to the important public health outcome from the inactivation of microbial pathogens in drinking water supplies, the demands for cost effective, large-scale provision of potable water have driven an expansion in the use of chlorination worldwide. Communities ranging in size from large urban centres to individual family homes in rural settings all require access to safe drinking water that is provided through a wide variety of distribution system types. These systems experience a range of operational issues that are often of a microbial character, such as persistence of pathogens and development of microbial biofilms for which disinfection or microbial control strategies are important [3].

In addition to drinking water, chlorination is frequently used for disinfection of water in swimming pools [4] and tertiary treatment of wastewater [1]. Increasingly however, evidence suggests that the residual byproducts of water disinfection when using chlorination, such as trihalomethanes, can have negative health effects [5–7]. In addition, environmental conditions can occur where chlorine is not effective in adequately disinfecting water, leading to risk of infection [2, 8, 9]. Alternative measures to chlorination or post-chlorination applications that mitigate the negative aspects could extend the usefulness of chlorine in providing safe drinking water.

Hydrogen peroxide (H_2_O_2_) has also been used for many years for water disinfection [2] and is generally considered to have low ecotoxicity as well as having no odor or colour [10, 11]. Oxidizing agents such as H_2_O_2_, also commonly known as reactive oxygen species (ROS), have also been extensively used as antiseptics however their lack of specificity to microbial cells over host/mammalian cells have tended to limit the use of H_2_O_2_ to topical therapeutic applications [12]. In order for H_2_O_2_ to be an effective microbicide it must be able to overcome the myriad of cellular mechanisms that are in place to deal with ROS that occur normally due to aerobic respiration, as well as inducible defence mechanisms that provide antioxidizing activities in the presence of exogenous oxidants, a situation termed oxidative stress. Bacterial pathogens have probably evolved ROS-responsive defence mechanisms to help deal with oxidative killing that is part of the host defence against invading microbes [12]. One common bacterial strategy to deal with excess H_2_O_2_ is to degrade H_2_O_2_ via enzymes collectively termed catalases; monofunctional catalases catalyze the disproportionation reaction while bi-functional catalases have both H_2_O_2_ degrading and H_2_O_2_ reducing activities [13]. In addition, organisms frequently contaminating water such as *E*. *coli*, produce several peroxidases such as NADH peroxidases, thiol peroxidase, cytochrome C peroxidase, bacterioferritin comigratory protein, glutathione peroxidase, and rubrerythrins, all to deal with H_2_O_2_ [13]. As little as 1 μM H_2_O_2_ can oxidize the cellular pool of free ferrous iron, thereby generating hydroxyl radicals that can cause significant DNA damage [14] via the Fenton reaction [15], therefore the pool of H_2_O_2_ scavenging enzymes is diverse.

Dental unit water systems, used to irrigate patients’ mouths during treatments, pose a significant water disinfection challenge as they are often contaminated with high numbers of bacteria, including opportunistic pathogens and organisms frequently forming biofilms. A recent study looked at combinations of silver and H_2_O_2_, as well as other compounds, to disinfect the dental units and found a silver/ H_2_O_2_ combination to be the most effective [16]. Early studies examining the biocidal effects of hydrogen peroxide combined with ionic silver clearly showed a synergistic killing effect against *Escherichia coli* [17, 18] while more recent studies have examined efficacy against a broader range of Enteriobacteriaceae [19] and organisms commonly found in swimming pools [20] while more recently silver is employed in nanoparticle formulations [21].

Silver, a biologically non-essential metal, has been investigated and used as a biocide for many years [22], where multiple strategies are being proposed for treatment of drinking water [23–25]. Treatment of drinking water systems with silver in combination with copper [26, 27] and of hospital hot water systems with a silver/hydrogen peroxide compound [28] to prevent Legionella has been shown to be efficacious. The World Health Organization deemed that up to 100 ug/L (ppb) silver could be present in drinking water without posing health risks [29]. The monovalent silver cation (Ag[I]) is the active species, interacting with amines, hydroxyls, phosphates and thiols via their nitrogen, oxygen and sulfur-containing electron donating groups both at bacterial membranes and within the cell [21, 22]. These interactions ultimately lead to disruption of the proton motive force, deregulation of the electron transport system and increased membrane permeability leading to cell death [21].

Huwa-San peroxide (HSP), a recently developed H_2_O_2_ that is stabilized with the addition of low concentrations of ionic silver (0.013–0.017%), is designed for application in the disinfection of water and hard surfaces, and veterinary-related uses [30]. The first North American site to employ hydrogen peroxide in drinking water (Killaloe Water Treatment Plant has been part of recent pilot studies approved by the Ontario Ministry of the Environment, and under the auspices of the Ontario Clean Water Agency, investigating the application of HSP as a secondary disinfectant. Unpublished data indicates that HSP can be successfully used as a secondary disinfectant, contribute to the significant reduction of trihalomethanes, and that residual levels of HSP remained throughout the distribution system. Although synergistic antimicrobial properties provided by a combination of H_2_O_2_ and ionic silver were previously demonstrated using silver nitrate to which H_2_O_2_ had been added [17–20], there are no published studies examining the biocidal properties or mechanism of HSP. While the biocidal mechanisms of H_2_O_2_ [31]or silver [21] are well studied, the synergistic mechanism of combined formulations of silver and H_2_O_2_ are poorly understood.

The aim of this study was to evaluate HSP against both Gram negative and positive bacteria and to examine the HSP mechanism. This study compares the disinfection efficacy of HSP, lab grade H_2_O_2_, and NaOCl minimum inhibitory concentrations for suspended (planktonic) cultures of indicator bacteria. The activity of HSP, H_2_O_2_, and the proprietary silver solution present in HSP were examined separately to evaluate the contribution of each component to bacterial killing. The results demonstrate that HSP, but not H_2_O_2_, preferentially interacts with the bacterial cell surface in a manner likely mediated by silver, thereby concentrating the biocidal activity of HSP at its target and increasing efficacy.

## Materials and Methods

### Strain and culture conditions

Laboratory strains of *E*. *coli* K12, *P*. *aeruginosa* PAO1, *B*. *subtilis* and *S*. *aureus* were acquired from Queen’s University’s microbiology unit strain collection. Environmental *E*. coli strains were collected in Kingston, Ontario by Dr. Aston of Queen’s University and were as follows: Lake Ontario water sample 1 (LOWS1), Lake Ontario water sample B6 (LOWS1-B6), Commodore’s Cove Lake 1 (CCL1), and Cataraqui Mall pond (CMP-B11A). The *E*. *coli* K12, *B*. *subtilis*, and environmental *E*. *coli* isolates were maintained on Luria Bertani (LB) agar while the *S*. *aureus* and *P*. *aeruginosa* PAO1 isolates were maintained on full strength Tryptic Soy agar (TSA). For starting inocula a single colony was picked from the respective agar plate, transferred to 50 mL of the appropriate growth medium and incubated for 17 hours with aeration (150 RPM shaking) at 37°C.

### Media and solution preparation

#### Media

Luria Bertani (LB) broth was prepared by dissolving 10 g of tryptone, 5 g of yeast extract and 5 g of sodium chloride (Fisher Scientific, Ottawa, ON, Canada) in a final volume of 1 L Millipore MilliQ (Millipore Canada, Toronto, ON, Canada) distilled and deionized water (MDD). Full strength tryptic soy broth (TSB) was prepared by dissolving 30 g of Bacto TSB Soybean-Casein Digest powder (BD Biosciences, Mississauga, ON, Canada) in a final volume of 1 L with MDD. The tap water used in this study was de-chlorinated by vigorously stirring 1 L volumes with a stir bar for a minimum of 16 hours. After de-chlorination, the tap water was adjusted to either pH 7.0 or 8.5 and filter sterilized using a 0.22 μm filter. The saline (0.9% NaCl), CaCl_2_ and KCl solutions (Fisher Scientific, Ottawa, ON, Canada) were all prepared as 1 M stock solutions and diluted to the appropriate working concentrations using MDD. Tris buffer (10 mM; Fisher Scientific, Ottawa, ON, Canada) was used to buffer the CaCl_2_ and KCl solutions at pH 7 and the tap water at pH 7.0 and 8.5. For the synthetic MIC, CT and cell-associated assay starting inoculums, a synthetic wastewater media [32] was prepared daily with a chemical oxygen demand (COD) of approximately 600 mg/L and a COD: Nitrogen: Phosphorus ratio of 100:5:1 [final concentrations of 250 mg/L glucose and 283.5 mg/L sodium acetate for the carbon sources, 8.78 mg/L KH_2_PO_4_, 11.24 mg/L K_2_HPO_4_ for the phosphorus source, 89.33 mg/L of (NH_4_)_2_SO_4_ for the nitrogen source, 5.07 mg/L MgSO_4_7H_2_O, 2.0 mg/L CaCl_2_2H_2_O, 0.01 mg/L NaMoO_4_2H_2_O, 0.36 mg/L MnCl_2_4H2O, 0.50 mg/L FeSO_4_7H_2_O, 0.39 mg/L CuSO_4_5H_2_O, 0.44 mg/L ZnSO_4_7H_2_O and 0.41mg/L Cl_2_6H_2_O for the trace metal sources,3.0 mg/L Fe(III) EDTA for the iron source and 100mg/L yeast extract]. The Fe (III) EDTA solution was prepared as per EDTA [33]. Agar plates were prepared by adding 15 g of granulated agar (Fisher Scientific, Ottawa, ON, Canada) to 1 L of the respective media.

#### Test products

Three oxidizing biocides were selected: laboratory grade 3% Hydrogen Peroxide (Fisher Scientific, Ottawa, ON, Canada), laboratory grade 5% Sodium Hypochlorite (RICCA Chemical Company, Arlington, TX, USA) and 19.9% HSP (SanEcoTec Ltd, Ottawa, ON, Canada). Two types of silver were tested. First, was the proprietary silver solution found in HSP, termed “HuwaSilver” (SanEcoTec Ltd, Ottawa, ON, Canada), which was received as a concentrated stock solution containing 363 ppm silver (concentration confirmed by Flame Atomic Absorption Spectroscopy (Agilent Fast Sequencer (FS) 280), Queen’s University Analytical Services). The second was certified ACS silver nitrate (Fisher Scientific, Ottawa, ON, Canada). All working solutions were prepared aseptically with sterile media immediately before conducting the experiments.

#### Neutralizer preparation

The catalase: peroxide ratio for successful neutralization of peroxide was determined to be at least 1:2. A stock solution of 3.4% bovine liver catalase (Sigma-Aldrich, St. Louis, MO, USA) was diluted to the appropriate working concentration aseptically in sterile test media. Sodium thiosulfate (Fisher Scientific, Ottawa, ON, Canada) was used for neutralizing hypochlorite as indicated by [34]. In the current study a 6% solution of sodium thiosulfate was used to ensure neutralization of the chlorine. After employing the neutralizers, chlorine and peroxide test strips were used to measure residual levels of biocide; a reading of 0 ppm indicated sufficient neutralization.

#### Sterility techniques used for the reagents

Ultrapure water (Millipore MilliQ, Millipore Canada, Toronto, ON, Canada), nutrient rich media, stock synthetic wastewater media components (except for Fe (III) EDTA), saline, KCl and CaCl_2_ were all sterilized by autoclaving. The Fe (III) EDTA, pH controlled de-chlorinated tap water, sodium thiosulfate and catalase were filter sterilized using a 0.22 μm filter.

### Minimum inhibitory concentration (MIC) assays

MIC assays determined the minimum concentration of disinfectant required to inhibit the overnight growth of planktonic bacteria [35]. A variety of different organisms were tested in nutrient rich media (TSB or LB broth) and synthetic wastewater media. The desired test medium was inoculated with a pure culture and incubated for 17 hours with aeration at 37°C. The same type of media was used in flat bottom 96 well microtitre plates (Sarstedt, Saint Léonard, QC, Canada) to determine the MIC. Initially, MIC ranges were determined using 2 fold successive dilutions of biocide to achieve the desired concentration range. Inocula of 10^6^ CFU/mL, standardized using a predetermined conversion factor relating CFU/mL to OD_600_, were prepared in 10 mL volumes and 50 uL was added resulting in a final inoculum concentration of 5 X 10^5^ CFU/mL. Positive growth controls (50 uL media +50 uL inoculum) and negative growth controls (100 uL media) were included. The microtitre plates were incubated in static conditions overnight at 37°C. Using a Varioskan (Thermo Scientific, Burlington, ON, Canada) spectrophotometer, the optical density (OD_600_) in each well was measured. An OD_600_ greater than 0.1 was considered to be positive growth. Once a preliminary MIC range was determined, subsequent MIC assays over narrower concentration ranges were conducted to determine a more accurate MIC.

### Contact/kill Time (CT) assays

CT assays determined the bacterial killing achieved after various durations of exposure to 20 ppm of disinfectants. The bacterial loads tested were 10^6^ CFU/mL and 10^3^ CFU/mL *E*. *coli* K12 in tap water (pH 7/8.5). Synthetic media was inoculated with 1 mL of pure culture (culture containing 1 genus/species only), and incubated for 17 hours with aeration at 37°C. To rinse the cells, aliquots of a 17 hour culture were spun down at 14,000 x g for 5 minutes and re-suspended in sterile de-chlorinated tap water. The rinsing process was repeated twice before taking the OD_600_ reading. Using a predetermined conversion factor relating CFU/mL to OD_600_, 10^3^ or 10^6^ CFU/mL cultures were prepared in 10 mL of sterile, de-chlorinated tap water buffered with 10 mM TRIS at the desired pH. Disinfectants were added to 10 mL cultures at 20 ppm with moderate shaking for the duration of the experiment. At multiple time points, an aliquot was removed and mixed with the neutralizing solution. The neutralized mixtures were serially diluted in de-chlorinated tap water and 100 uL of each dilution was plated on LB agar; the plates were incubated for 17 hours at 37°C. The CFU/mL were calculated by counting plates with between 5–300 colonies from the various time points and multiplying by the required dilution factor.

#### Neutralization of biocides

Assays were designed to determine if the biocide was being completely neutralized during the CT assays. As per the time-kill assays, 10^3^ and 10^6^ CFU/mL *E*. *coli* K12 cultures were prepared in sterile tap water at pH 7 and exposed to 20 ppm HSP or peroxide. At the desired time points, four 500 uL aliquots were removed and spun down at 14, 000 X g for 5 minutes. Following centrifugation, 450 uL of supernatant was removed and discarded. Two of the pellets were re-suspended in 450 uL of sterile, de-chlorinated tap water (pH 7) and the other two pellets were re-suspended in 450 uL of 20 ppm sterile catalase. These were serially diluted in sterile de-chlorinated tap water (pH 7) and 100 uL of each dilution was plated on LB agar. The plates were incubated for 17 hours at 37°C. CFU/mL were calculated by counting plates with between 5–300 colonies from the various time points and multiplying by the required dilution factor.

#### Determining the effect of KCl and CaCl2 on the killing efficacy

CT assays were performed in KCl and CaCl_2_ solutions to determine if the presence of mono and divalent ions in solution would influence the killing efficacy of HSP and H_2_O_2_. The experiments were conducted exactly as previous CT assays, however in addition to testing in tap water, solutions of 0.005, 0.015, 0.025 and 0.05 M KCl and CaCl_2_ were used. Tris buffer (10 mM, pH7) was included. After 60 minutes of contact time, catalase neutralizing solution was added, cultures were serially diluted and 100 uL of each dilution was plated on LB agar. The plates were incubated for 17 hours at 37°C. The CFU/mL values were calculated by counting plates with between 5–300 colonies and multiplying by the required dilution factor.

### Carbon dioxide measurement

A Continuous Emissions Monitoring System (CEMS) for measuring carbon dioxide was constructed as described by [36]. A silicone tube biofilm reactor (inside diameter, 0.16 cm; outside diameter, 0.24 cm; length, 150 cm; VWR International, Mississauga, ON, Canada) was encased in a sealed Tygon tube (inside diameter, 0.48 cm, outside diameter, 0.79 cm; formulation R-3603; VWR International, Mississauga, ON, Canada). The annular space of the CEMS was connected to an absolute, nondispersive, infrared LI-820 CO_2_ gas analyzer (LI-COR Biosciences, Lincoln, NE, USA) and CO_2_ free compressed air (Linde Canada Limited, Kingston, ON, Canada) was used as a sweeper gas. Gas flow rates were controlled by an HiQ Baseline brass 1-stage regulator with an NPT female outlet (Linde Canada Limited, Kingston, ON, Canada) and a thermal gas mass flow meter set to 9.5 mL/min (Aalborg, NY). The Li-820 CO_2_ gas analyzer was calibrated using a two-step calibration process. First, the analyzer was zeroed by running the CO_2_ free compressed air through the analyzer and manually setting the CO_2_ reading to 0 ppm. Subsequently, an 1800 ppm CO_2_/air mixture (Linde Canada Limited) was passed through the system while the CO_2_ reading was manually set to 1800 ppm. The 1800 ppm CO_2_/air calibration gas’s flow rate was controlled using the previously mentioned HiQ Baseline regulator.

#### Biofilm

After assembly of the CEMS, the apparatus was disinfected with 0.525% NaOCl in dH_2_O for a minimum of 2 hours. Subsequent overnight irrigation (minimum 16 hours) with dH_2_O rinsed the bleach from the tubes. Prior to inoculation, sterile 1% TSB was flushed through the tubes for 30 minutes to displace the dH_2_O. As per a modified version of Kroukamp and Wolfaardt’s [36] procedure, pure culture biofilms of *P*. *aeruginosa* PAO1 were grown in the silicone tubes. Using a sterile needle and syringe, the silicone tubes were inoculated with 200 μL from an overnight culture grown in 10% TSB; the pump was turned off for 30 minutes to allow initial adhesion to the tube walls. After adhesion, sterile 1% TSB was continuously supplied by a Masterflex L/S peristaltic pump (Cole Parmer Canada Inc., Montreal, QC, Canada) at 21 mL/min. All biofilms were developed until CO_2_ levels indicated a steady metabolic state was achieved for 20 hours at 25°C using a water bath to control the temperature.

#### Biocide treatment of biofilms

After the biofilm reached a steady metabolic state, the inflowing medium was switched to freshly prepared HSP, HuwaSilver or silver nitrate solutions in 1% TSB. The tested HSP concentrations were 70, 100, 140 and 500 ppm, which respectively contained HuwaSilver concentrations of 50, 75, 100 and 375 ppb. Tested molar concentrations of silver from silver nitrate matched the HuwaSilver concentrations. The duration of each constant flow exposure was 2 hours; post exposure, the inflowing medium was switched back to 1% TSB and the biofilms initiated their recovery process. Throughout the exposure and recovery process, fluctuations in CO_2_ production rates were monitored. Once the biofilm recovered to a steady metabolic state for 20 hours after the initial exposure a second treatment with the same biocide was carried out. After the second exposure was complete, the CEMS was sterilized and a new biofilm was initiated as described in section 2.11.

#### Viable cell counts and live/dead cell imaging

Approximately 1.5 mL of effluent was collected from the disconnected downstream tubing at time = 0 hours (once the biofilm achieved a steady metabolic state for 20 hours) and at time = 2 hours (once the 2 hour exposure to the test solution concluded). The effluent was spun down at 14,000 X g for 5 minutes and re-suspended in sterile 0.9% NaCl twice. The effluent was serially diluted in sterile 0.9% NaCl and plated on 10% TSA. After 24 hr incubation at 37°C CFU/mL values were calculated by counting plates with between 5–300 colonies and multiplying by the required dilution factor.

A modified version of a viable cell count procedure [36] was carried out and additional effluent was collected and washed for epifluorescent microscopic imaging. Using a live/dead BacLight Bacterial Viability Kit (Invitrogen Canada, Burlington, ON, Canada), bacterial viability was determined as per the manufacturer’s protocol. Prepared slides were examined with 40 X magnification at the objective using a confocal microscope (Zeiss LSM710, Carl Zeiss Canada, Toronto, ON, Canada) equipped with an Argon 488 laser. The number of live and dead cells per microscope field was analyzed using the image analysis software provided by the manufacturer and live:dead ratios were calculated.

## Results

### Minimum Inhibitory Concentrations (MICs) of biocides

A panel of Gram negative or positive laboratory and environmental isolates was selected to compare the MICs for HSP, H_2_O_2_, and NaOCl. These results are not meant to indicate absolute MIC values for each biocide/organism, but rather are a means to compare biocide susceptibility under a defined set of conditions as it is well established that MICs can vary significantly depending upon inoculum size, media, and the length of incubation [37]. Table 1 summarizes data comparing MICs in either rich media (tryptic soy broth or Luria broth) or synthetic wastewater media (SYN) for selected test organisms. Bacteria showed greater susceptibility to all biocides in SYN versus rich media. Environmental isolates of *E*. *coli* were slightly more susceptible to HSP or H_2_O_2_ than the K-12 laboratory strain, however some environmental isolates had a higher NaOCl MIC than the K-12 strain. When comparing the susceptibility to HSP and H_2_O_2_ MICs were very similar, with *S*. *aureus* being the most sensitive, followed by *P*. *aeruginosa*, *E*. *coli* and then *B*. *subtilus* over a range of 8 to 59 ppm biocide in rich medium. Of these 4 strains *P*. *aeruginosa* was the most resistant to NaOCl.

**Table 1:** Minimum inhibitory concentration (MIC) range of biocides for microorganisms tested in nutrient rich and synthetic wastewater media (10^5^ CFU/mL inoculum).

		Minimum inhibitory concentration (ppm)		
Bacteria	Media^1^	HSP	H_2_O_2_	NaOCl
*P*. *aeruginosa* PAO1	TSB	24–40	32–40	640
	SYN	10	15	80
*B*. *subtilis*	LB	59	59	400
	SYN	25	25	30–70
*S*. *aureus*	LB	8	8	309–463
	SYN	10–25	10	50–90
*E*. *coli* K12	LB	39–49	39–59	294–412
	SYN	10–15	10–15	30–50
*E*. *coli* CMP-B11A	LB	24–30	24–30	350
*E*. *coli* CCL1	LB	32	24–32	300–400
*E*. *coli* LOWS1	LB	32–40	32–40	350–400
*E*. *coli* LOWS1-B6	LB	25–40	30–40	294–412
*E*. *coli* KS1	LB	30–36	42	480
*E*. *coli* KS2	LB	36	36	320

^1^TSB—Tryptic soy broth; LB- Luria Bertani; SYN—synthetic wastewater media

### Killing efficacy of biocides is pH and bacterial cell density dependent

**Fig 1:**
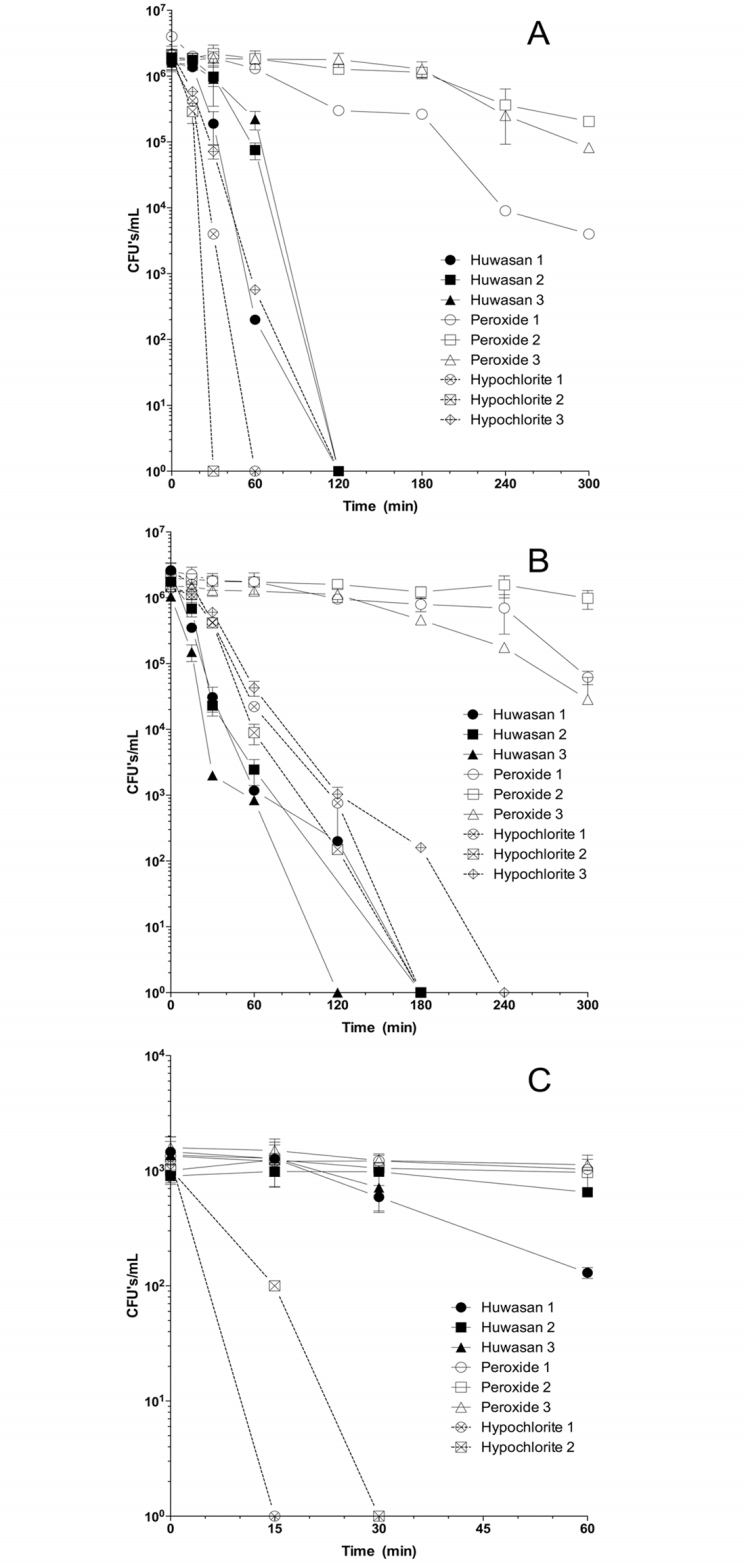
Comparison of pH and cell density dependent killing efficacy of HSP, H_2_O_2_, and NaOCl Killing efficacy of biocides (20 ppm) at pH 7.0 on 10^6^ *E*. *coli* (A), at pH 8.5 on 10^6^ *E*.*coli* (B), and at pH 7.0 on 10^3^ *E*. *coli* (C) as measured by total CFU/mL over 300 min (A,B) or 60 min (C). H_2_O_2_ is much slower at killing bacteria at pH 7.0 than either HSP or HOCl_2_ (A). Raising the pH to 8.5 increases the 100% kill times of NaOCl to equal to or greater than that of HSP (B). At lower cell densities HOCl_2_ reaches 100% killing in 15–30 minutes while both HSP and H_2_O_2_ show slower killing kinetics (C).

In order to determine the ability of the biocides to kill the test bacteria in a time dependent manner *E*. *coli* were treated with the same concentration (20 ppm active) of each biocide and, at multiple time points, the number of viable cells was determined after neutralization of the biocide (Fig 1). To determine the effect of pH on killing efficacy, killing time at pH 7.0 versus 8.5 was examined (Fig 1A and 1B). At pH 7.0 all bacteria were killed with either HSP or NaOCl within 120 minutes, whereas exposure to H_2_O_2_ resulted in a 1–2 log reduction in 300 minutes. At pH 8.5, killing efficacy by H_2_O_2_ was reduced to slightly over 300 minutes of exposure, while the time for complete killing by NaOCl increased to 180–240 minutes and HSP increased slightly to 120–180 minutes.

The effect of bacterial density was tested by comparing the killing efficiency of 10^6^ cells/mL with 10^3^ cells/mL. Killing of 10^3^ bacteria with NaOCl reached 100% by 15–30 minutes, a slightly higher efficiency than with 10^6^ cells. H_2_O_2_ killing efficiency was the same with 10^3^ and 10^6^ bacteria within 60 minutes. Unpredictably, at 60 minutes less than 1 log reduction was achieved with HSP using 10^3^ bacteria compared with approximately a 3 log order reduction of culturable cells when starting with 10^6^ bacteria. Contrary to expectations, based on the ratio of bacteria to biocide molecules where it was anticipated that fewer bacteria would be killed faster under the same parameters, HSP performed differently. After removing bacteria from the HSP solution by centrifugation, HSP killing kinetics were inverse to expectations, with higher bacterial concentrations being killed more efficiently. This result prompted an examination of the effect of neutralization of the biocide on kill times at the two bacterial concentrations with HSP versus H_2_O_2_.

### Differences in susceptibility of HSP and H_2_O_2_ to neutralization by catalase

**Fig 2:**
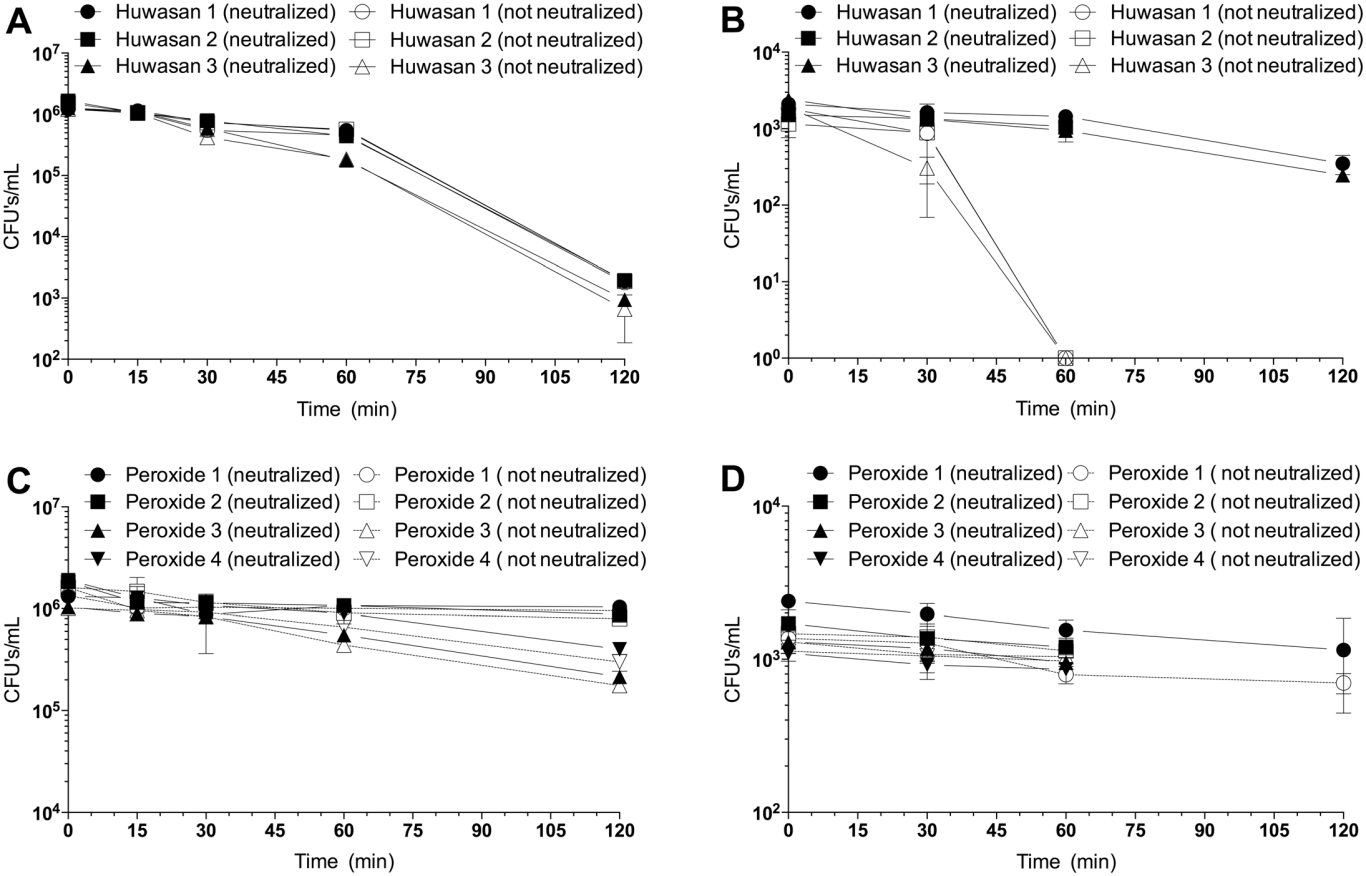
Comparison of the effect of neutralization of biocide on killing of *E*.*coli* at high and low cell densities. Killing efficacies of HSP or H_2_O_2_ (20 ppm) was measured at high (A, C) or low (B, D) cell densities with and without neutralization of biocide by addition of 20 ppm catalase. Killing rates at high cell densities by HSP or H_2_O_2_ are not influenced by neutralization (A, C), whereas at low cell densities bacteria exposed to HSP, but not neutralized, are killed significantly more quickly than those neutralized (B). This effect is not seen with H_2_O_2_ at the low cell densities (D).

Neutralization of the biocides was being carried out in order to quickly stop the killing effect of the biocides while the bacteria were being prepared for plating by pelleting the bacteria, discarding the biocide-containing supernatant and diluting the bacteria for determination of CFU. Preliminary experiments had shown that to halt killing it was insufficient to remove bacteria from the biocide solution, especially when testing HSP (data not shown). Any biocide associated with the bacterial cell would remain with the cell after centrifugation. When the killing rate of 10^6^ bacterial cells was examined with or without neutralization there was no difference with either HSP or H_2_O_2_ although HSP was more effective at killing than H_2_O_2_ as previously shown (Fig 2A and 2C). There was little killing by H_2_O_2_ using 10^3^ bacterial cells and no difference in the presence or absence of neutralization (Fig 2D). When 10^3^ cells were tested with HSP, there was a significant difference in killing rate in the presence and absence of neutralization, with the bacteria being killed much more quickly in the absence of neutralization (Fig 2B). This result suggested that HSP was interacting with bacterial cells differently than H_2_O_2_. The data suggested that at bacteria (cells): HSP(ppm) ratios of 10^6^:20 most of the HSP was bound to the bacterial cell surface and neutralization was inefficient. Thus, when the bacterial concentrations were lower, neutralization was likely more efficient owing to the higher levels of disinfectant in solution. This is further supported by the observation that, in the absence of neutralization, significant levels of active biocide remained associated with the bacterial cells after centrifugation (Fig 2B). H_2_O_2_ however, did not show the same killing kinetics (Fig 2D). When the bacteria were separated from the soluble H_2_O_2_ via centrifugation, killing almost completely ceased suggesting that once the soluble H_2_O_2_ is removed or neutralized, there is little to no cell associated effect.

### Effect of mono and divalent cations on biocide killing efficacy

The initial interaction of HSP with the bacterial cell surface could be of an electrostatic nature due to the presence of positively-charged silver ions. It was predicted that the addition of mono or divalent cations to the biocide solution would compete with HSP, but not H_2_O_2_, and alter the killing efficacy. It was demonstrated that KCl and CaCl_2_ solutions from 0.005 to 0.05 M showed a significant ability to inhibit killing of *E*. *coli* by HSP while these salts had no effect on H_2_O_2_-mediated killing. In addition, the magnitude of inhibition by the divalent CaCl_2_ was greater than that of monovalent KCl (Table 2). These data support bacterial cell surface interactions as an important step in the mechanism of HSP killing that is not seen with H_2_O_2_ and therefore attributable to the silver present in HSP.

**Table 2:** Effect of mono and divalent salt concentrations on the killing efficacy of 20 ppm HSP or H_2_O_2_ against 10^6^ CFU/mL *E*. *coli* K12 at pH 7.

		Percent killing				
		Salt concentration (M)				
	Salt	0	0.005	0.015	0.025	0.05
**HSP**	KCl	100	85	76	70	84
	CaCl_2_	100	58	60	43	45
**H_2_O_2_**	KCl	100	100	94	91	100
	CaCl_2_	100	100	100	100	100
**No Biocide**	KCl	0	1	0	3	3
	CaCl_2_	0	3	5	1	2

All values are normalized to killing in the absence of cations.

### Comparison of silver-mediated antimicrobial activity on biofilms

**Fig 3:**
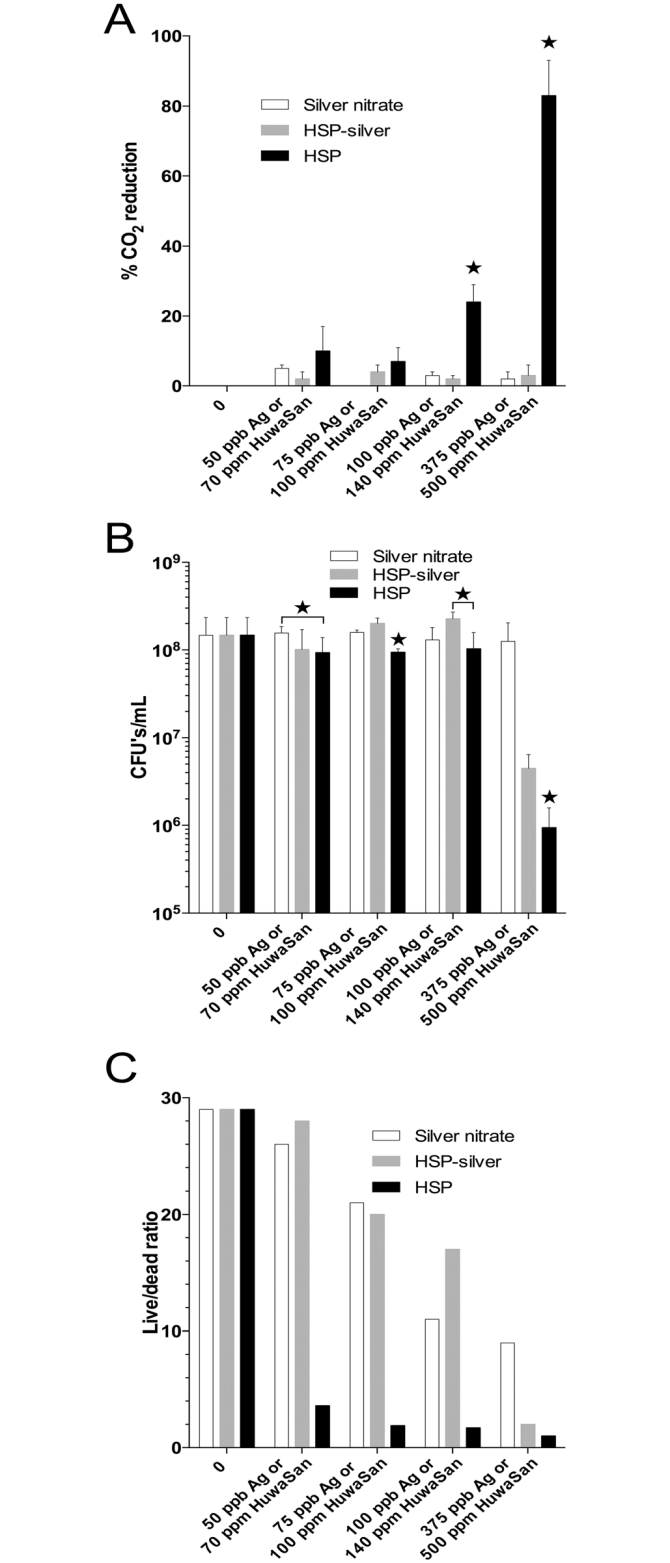
Comparison of HSP, HSP-silver and silver nitrate on the metabolism and cell viability of a *P*. *aeruginosa* biofilm in a continuous flow apparatus. Stable, established biofilms of *P*. *aeruginosa* monocultures were exposed to the indicated concentrations of HSP, HSP-silver or silver nitrate for 2 hrs and CO_2_ generated was monitored and plotted as the maximum reduction relative to the untreated biofilm pre biocide exposure (A). While silver nitrate and HSP-silver had negligible effects on metabolism, HSP treatment of the biofilm significantly inhibited metabolism. (B) and (C) show the viability of planktonic cells released from the biofilm measure by total viable count (B) or by enumerating live and dead bacteria via epifluorescence microscopy using the BacLight Bacterial Viability Kit (C). Treatment with HSP shows a dose dependent effect on cell viability at more significant levels than either HSP-silver or silver nitrate. Stars indicate HSP treatments statistically significantly different from silver treatments (P ≤0.05) Bars are included where HSP treatment is significantly different from only one type of silver treatment.

To distinguish the effect of silver and H_2_O_2_ on the metabolic activity of a *P*. *aeruginosa* PAO1 biofilm, a comparison of the antimicrobial activity of HSP was carried out with either silver formulated in the same manner as in HSP but not associated with H_2_O_2_ (HuwaSilver), as well as silver supplied in the form of silver nitrate. After establishment of a stable biofilm within a continuous flow cell, it was treated with several concentrations of HSP and corresponding concentrations of ionic silver over the range of 70–500 ppm and 50–375 ppb, respectively, and the reduction in metabolic activity, measured by CO_2_ levels, was monitored (Fig 3A). Increasing concentrations of HSP showed a dose dependent effect reaching an 83% reduction in CO2 emission at the highest concentration used (500 ppm). HuwaSilver or silver nitrate were found to have no, or a negligible, effect on metabolic activity over two hours of continuous exposure (Fig 3A).

Additional measures of biocide/silver activity were obtained by quantifying the number of viable cells (Fig 3B) and by calculating the ratio of live to dead bacteria released into the bulk liquid from the CEMS effluent (Fig 3C). After exposure to the previously described concentration range, HSP reduced the number of viable cells by 37% to 99% with increasing concentrations of biocide compared to 12% (100 ppb) or 15% (375 ppb) for silver nitrate and 33% (75 ppb), 37% (100 ppb) or 97% (375 ppb) for HuwaSilver. Furthermore, direct fluorescent microscopic counts showed a HSP concentration dependent decrease similar to the results from recovering culturable bacteria to a low of a 1:1 live:dead cell ratio at the highest biocide concentration. Live:dead ratios of cultures treated with either silver nitrate or Huwa-silver maintained much higher viability levels, although HuwaSilver treated cultures had a live:dead ratio of only 2 at the highest silver concentration.

## Discussion

The three biocides that were chosen for comparison in this study are commonly used for water disinfection where they affect microbial viability via the production of reactive oxygen species (ROS) that cause damage to enzymes and DNA within the bacteria cell [38]. Since ROS, such as superoxide (O^-^_2_), H_2_O_2_, and hydroxyl radicals (HO) are constantly generated as a result of the normal metabolism of oxygen, both prokaryotic and eukaryotic organisms have evolved a complex array of mechanisms to sequester and inactivate low levels of ROS [38, 39]. Biocide concentrations must therefore be maintained at levels higher than can be accommodated by the target organisms in order to be effective. Biocide concentrations applied must also take into account the impact of various environmental conditions that may compromise the efficacy of the biocide.

The most active aqueous species formed by dissolution of NaOCl in water, hypochlorous acid (HOCl), is predicted to cause irreversible unfolding and aggregation of bacterial cellular proteins, especially thermolabile proteins, which ultimately leads to cell death [40]. NaOCl has been shown to be more potent than most other known oxidants but this potency is highly affected by environmental conditions such as pH and the presence of inorganic and organic molecules [41]. This effect was demonstrated in both the MIC determinations (Table 1) and the kill time tests (Fig 1) using NaOCl. Synthetic wastewater media, which contains a much lower level of organics than Luria Bertani or tryptic soy broth, promoted a much higher efficacy of NaOCl killing than rich media with all bacteria tested. A higher bacterial load, which can be considered a higher organic load, also increased the killing time of NaOCl when killing of 10^6^ CFU/mL was compared to killing of 10^3^ CFU/mL (Fig 1C) of *E*. *coli*. At a pH of 7.0 to 8.0, hypochlorous acid dissociates into the much less effective hypochlorite ion (OCl^-^), thereby decreasing the killing efficacy [41]. The kill time tests carried out at pH 7.0 and pH 8.5 (Fig 1A and 1B) showed increased kill times of *E*. *coli* with NaOCl at the higher pH, reflecting a predicted loss in biocidal activity at the higher pH. When comparing the NaOCl susceptibility of the various organisms tested, *P*. *aeruginosa* required the highest NaOCl concentrations, *B*. *subtilis* and *S*. *aureus* fell in the middle with *E*. *coli* requiring the lowest, although some environment *E*. *coli* strains showed lower susceptibility to NaOCl mediated killing. This general pattern of bacterial sensitivity to NaOCl follows that demonstrated previously [42].

While still a highly effective disinfectant of surfaces, chlorination of drinking water and the subsequent generation of residuals, such as trihalomethanes among a long list of disinfectant by-products [5], have become a significant concern due to their toxicity and mutagenicity [4–7, 43, 44]. New approaches to water disinfection are called for. In addition to being a biocide, peroxide, via the combination of H_2_O_2_ and Fe^2+^ known as the Fenton reaction [15], can also be used to reduce the levels of some of the problematic by-products of chlorine treatment of water [45, 46]. As a biocide H_2_O_2_ can oxidize, via the Fenton reaction, iron-sulphur clusters in a number of bacterial dehydratases, thereby inactivating them causing cell death [38]. H_2_O_2_ is relatively small and uncharged and is thought to cross membranes as efficiently as water. Endogenous concentrations of H_2_O_2_ are low, such that at extracellular H_2_O_2_ concentrations of 0.2 μM or greater, H_2_O_2_ will tend to move into the cell [47]. While the concentration of 20 ppm (~600 μM) H_2_O_2_ used in the current study would therefore be toxic to bacteria, most organisms have developed a complex array of oxidative stress responses that result in the upregulation of ROS scavenger molecules such as peroxidases and catalases that degrade H_2_O_2_ in an attempt to protect the cell [38].

A comparison of the MICs determined for both H_2_O_2_ and HSP show them to be very similar for all the bacteria tested (Table 1). These MICs are not identical from species to species however, which at least in part, reflects the ability of a particular bacterium to produce catalase. *P*. *aeruginosa* [48–50], *B*. *subtilis* [51], and *E*. *coli* [13, 52, 53], produce multiple catalases and hydroperoxide reductases, while *S*. *aureus* produces one of each [54]. For example, *E*. *coli* is proposed to synthesize at least nine enzymes that act as catalases or peroxidases [13]. This may account for the greater sensitivity to HSP and H_2_O_2_ of *S*. *aureus* compared to other tested bacteria. The wide variety of experimental methods commonly used to compare sensitivity to biocides makes comparison with previous results difficult however trends were similar [18–20] to those reported here. There also appears to be a difference in sensitivity to HSP and H_2_O_2_ between the MIC values and contact/kill time testing that used 20 ppm biocide (Table 1, Fig 1). Since the MIC testing was an endpoint determination with a 16 hr incubation time in the presence of biocide, it is likely that a small number of bacteria survived higher biocide levels by mounting a protective oxidative stress response upon initial exposure to biocide and then as the biocide levels decreased due to inactivation by catalase production over 16 hrs, bacteria were able to collectively recover similar to experiments reported by Davoudi et al.[19].

It was also of interest to determine if HSP displayed activity against bacteria growing in a biofilm. Bacterial biofilms are an important target of biocides used for water purification as biofilms represent an often difficult to treat mode of bacterial growth [9]. *P*. *aeruginosa* biofilms established within a continuous flow cell model system [36, 55, 56] were used here to test biocide efficacy by monitoring metabolic activity while being exposed to varying concentrations of HSP and silver. HSP, the form of silver present in HSP (HSP-silver), and silver from silver nitrate, were compared at equal silver concentrations. The metabolic activity of the biofilm, as measured by CO_2_ generation, clearly showed a significant inhibition by HSP, albeit at higher concentrations than previously seen to be effective for planktonic cells (Fig 3A). Concentrations of 70–140 ppm HSP decreased cell viability by 33–37% (Fig 3B) or resulted in live:dead ratios of 3.6–1.7 (Fig 3C) and even greater killing at 500 ppm HSP. Biofilms were exposed to biocide for only 2 hrs in these experiments and it is predicted that longer exposures would be needed to eliminate the biofilm. Others have shown an 85% reduction in *P*. *aeruginosa* biofilms with 1% H_2_O_2_ alone [57] In the field, biofilms are made of mixed microbial communities [58–61] and it is well established these adherent communities are more resistant to disinfection than planktonic bacteria [58, 61]. Treatment of established biofilms with H_2_O_2_ and H_2_O_2_/silver combinations has been shown to alter the total numbers of organisms within a biofilm [62] and to also significantly alter the diversity of organisms [63] but this study did not quantify the organisms present after different biocide treatments.

The kill curves generated with 10^6^ CFU/mL *E*. *coli* in the presence of HSP and H_2_O_2_ showed a pronounced difference indicating that HSP was much more effective than H_2_O_2_ (Fig 1A and 1B). Several possibilities exist for this difference. It has been proposed that addition of silver to H_2_O_2_ serves to stabilize H_2_O_2_, allowing higher efficacy through increased residual levels of H_2_O_2_ [18, 20, 62]. It may be that HSP is not as effective in inducing an oxidative stress response as H_2_O_2_ and, therefore, there are higher levels of catalase secreted in response to H_2_O_2_ that serve to neutralize it. The oxidative stress response occurs very quickly upon exposure to H_2_O_2_ and induces high levels of catalase [47, 64]. The transcriptional regulator, OxyR, of the H_2_O_2_-induced stress response system is activated within 30 s of exposure to H_2_O_2_ and is therefore able to rapidly enhance transcription of enzymes such as catalase [64, 65]. Pedahzur et al. [18] measured gene expression using promoter-*lux* fusions, finding that the OxyR regulon member for catalase (*katG*) was induced 40-fold in the presence of H_2_O_2_ but not at all in the presence of silver or H_2_O_2_+silver, suggesting that the silver blocked H_2_O_2_-mediated OxyR induction. Alternatively, the silver component of HSP may enhance its activity over that of H_2_O_2_, either in providing additional biocidal activity or in altering the mechanism by which HSP interacts with and enters the cell. Of the promoters tested by Pedahzur et al., only *grpE* and *dnaK*, both components of the heat shock response, were found to be synergistically induced in the presence of H_2_O_2_+silver [18]. These authors posit that the synergistic activity of H_2_O_2_+silver results from damage to proteins, which then increases expression of heat shock proteins responsible for folding and/or refolding cellular proteins; the specific contribution of protein damage to biocidal activity is unknown.

In contrast to the expected differences in response between HSP and H_2_O_2_, the observed differences in response between different cell densities (10^3^ and 10^6^ CFU/mL) with HSP but not with H_2_O_2_ were not expected (Fig 1C). It was predicted that given the higher ratio of biocide to bacteria at low cell densities, *E*. *coli* would be killed faster at the lower cell densities with HSP, not slower. Indeed, faster killing was observed with NaOCl. In these experiments, cell aliquots were removed from the biocide-containing solution and excess catalase was quickly added in order to neutralize and prevent carry-over of biocide while the bacteria were being prepared for plating. It appeared that biocide neutralization was better at stopping killing at low cell concentrations, suggesting that the accessibility of HSP by catalase was being affected. Experiments were repeated with the addition of a centrifugation step in order to quickly separate the bacteria from the bulk of the biocide in solution followed by +/- catalase treatment of the cell pellets (Fig 2). It was hypothesized that HSP was binding to the bacterial cell surface and enhancing its killing efficacy. If this was the case, pelleting the cells away from HSP in solution would not stop the biocidal effect of the HSP, but subsequent addition of catalase to the cell pellets would. This time at low cell densities HSP-mediated killing was rapid in the absence of neutralization by catalase, suggesting that HSP was indeed sequestered by the bacterial cell. This HSP interaction appeared to be at the bacterial cell surface as the cell-associated HSP could be quickly neutralized by the addition of catalase. At high cell densities killing with HSP was the same, regardless of the addition of catalase, never reducing the bacterial population to zero (Fig 2A). This was likely a combination of less HSP available per bacterium and less effective dispersion of catalase at such high cell densities. Similar experiments using H_2_O_2_ showed no difference in killing efficacies suggesting the silver component of HSP promotes a unique interaction between HSP and the bacterial surface (Fig 2C and 2D). These experiments point to differences in the biocidal mechanism of HSP compared to H_2_O_2_.

It was hypothesized that the positively charged silver ions in HSP were attracted to the bacterial cell surface through electrostatic interactions and that this interaction would be inhibited by the addition of excess cations. To test this, *E*. *coli* were exposed to either HSP or H_2_O_2_ in the presence of increasing amounts of KCl or CaCl2. These cations were able to inhibit HSP-mediated killing, but had no effect on H_2_O_2_-mediated killing. As well, the divalent cation was more inhibitory than KCl (Table 2). While it is well known that insoluble AgCl can be formed in the presence of Cl^-^, much higher concentrations of Cl^-^ than used here are necessary to precipitate silver [66]. Others have shown that metal ions such as copper decrease toxicity to silver in *E*. *coli*, the proposed mechanism being competition for sites at the bacterial cell surface [67]. A more recent series of experiments examining silver ion uptake by Holt and Bard [68] showed that in the presence of 1 μM AgNO_3_ approximately 40% of the silver binds to the outside of the cell, while the remainder is transported into the cell. Studies examining the effect of divalent cations on silver nanoparticle biocidal activity showed that a cation-dependent increase in nanoparticle size may have enhanced interaction with Gram negative bacteria [69]. The silver formulation in HSP does not display this enhanced activity. Although it was not determined in the current study if the added cations were directly associating with bacterial targets of silver ion interaction, the results clearly support the idea that silver in HSP promotes association of HSP with the bacterial cell.

Experiments were carried out to further explore if the silver component of HSP has biocidal activity in addition to that provided by the H_2_O_2_. In the continuous flow system used, there is a flux of bacteria at the surface of the biofilm as bacteria settle onto the surface or leave the biofilm as planktonic cells [55]. Sampling of the planktonic population at the conclusion of treatment showed a dose dependent killing effect that was significantly less with either HSP-silver or silver nitrate compared to HSP, although reductions of 12–15% in CFU’s and effects on live/dead ratios were detectable at 75–375 ppb silver (Fig 3). Silver nitrate solutions at concentrations of 100 ppb have previously been shown to inactivate planktonic *P*. *aeruginosa*, although significant killing required exposure of at least 8 hours at 24°C [26]. The toxicity of Ag^+^ is proposed to be due to several mechanisms. Several studies have shown that exposure of *E*. *coli* to silver nitrate results in a transient stimulation of respiration that is a consequence of the uncoupling of respiration from ATP synthesis [21, 68, 70, 71]. Studies have pointed to NADH dehydrogenase as the target of silver ion damage [21, 68], although additional cytoplasmic membrane protein targets are highly likely. Ultimately the induced increase in respiration results in superoxide and hydroxyl radical formation that further damages the cell [21, 68, 70]. Alteration of microbial proteins, both at the cell surface and internally has also been proposed [72], and is supported by early observations of silver-mediated induction of the heat shock response [18]. In order to demonstrate silver toxicity, many studies carried out by others used concentrations of ionic silver that were substantially higher than that used in the current study [19, 22, 67]. Given the low concentration of silver in HSP, unless the concentration of HSP in use is 500 ppm or higher, the results presented here indicate that the majority of the biocidal effects will be mediated through a synergism between H_2_O_2_ and silver activity rather than silver toxicity.

This study has conclusively shown that HSP displays significant biocidal activity towards several Gram positive and negative bacteria that are capable of producing significant levels of peroxide-neutralizing catalase. While the exact mechanism of HSP killing is still unknown, the data suggests that the silver component of HSP helps target HSP to the bacterial cell surface, thereby creating high local concentrations of H_2_O_2_. The mechanism of action of combinations of silver and H_2_O_2_ have not been clearly elucidated for either ionic or nanoparticle forms of silver [19]. It has been proposed that the addition of silver helps to stabilize the highly labile H_2_O_2_, thereby enhancing the residual levels of H_2_O_2_ and this effect has been noted with HSP (unpublished data). Several studies as discussed above noted a synergistic effect of combined silver and H_2_O_2_. It is unlikely that this synergism is a chemical effect of silver on H_2_O_2_ as the addition of ionic silver has also potentiated the activity of a variety of antimicrobials [73, 74] or metals [18]. While it has been demonstrated in this study that HSP associates with the bacterial cell surface to a greater extent than H_2_O_2_, the specific mechanism contributing to HSP efficacy remains to be determined. In addition, further studies such as those directed at understanding how organics and inorganics impact HSP efficacy in the field, as well as determining mechanisms mediating reduction of chlorination byproducts will significantly add to the knowledge base relating to the use of HSP in water disinfection.
